# Epigenomics and Early Life Human Humoral Immunity: Novel Paradigms and Research Opportunities

**DOI:** 10.3389/fimmu.2020.01766

**Published:** 2020-09-02

**Authors:** Maria J. Gutierrez, Gustavo Nino, Xiumei Hong, Xiaobin Wang

**Affiliations:** ^1^Division of Pediatric Allergy, Immunology and Rheumatology, Department of Pediatrics, Johns Hopkins University School of Medicine, Baltimore, MD, United States; ^2^Division of Pediatric Pulmonary and Sleep Medicine, Children's National Medical Center, George Washington University, Washington, DC, United States; ^3^Center for Genetic Medicine, Children's National Medical Center, Washington, DC, United States; ^4^Department of Population, Family and Reproductive Health, Center on Early Life Origins of Disease, Johns Hopkins Bloomberg School of Public Health, Baltimore, MD, United States; ^5^Division of General Pediatrics and Adolescent Medicine, Department of Pediatrics, Johns Hopkins University School of Medicine, Baltimore, MD, United States

**Keywords:** epigenomics, systems immunology, early life, antibodies, infancy

## Abstract

The molecular machinery controlling immune development has been extensively investigated. Studies in animal models and adult individuals have revealed fundamental mechanisms of disease and have been essential to understanding how humans sense and respond to cellular stress, tissue damage, pathogens and their environment. Nonetheless, our understanding of how immune responses originate during human development is just starting to emerge. In particular, studies to unveil how environmental and other non-heritable factors shape the immune system at the beginning of life offer great promise to yield important knowledge about determinants of normal inter-individual immune variation and to prevent and treat many human diseases. In this review, we summarize our current understanding of some of the mechanisms determining early life antibody production as a model of an immune process with sequential molecular checkpoints susceptible to influence by non-heritable factors. We discuss the potential of epigenomics as a valuable approach that may reveal not only relevant gene-environment interactions but important clues about immune developmental processes and homeostasis in early life. We then highlight the novel paradigm of human immunology as a complex field that nowadays requires a longitudinal systems-biology approach to understand normal variation and developmental changes during the first few years of life.

## Introduction

Mechanistic studies conducted over the past three decades have defined the basic molecular machinery that controls the development of protective immune responses in different cell populations (1–3). The use of animal models has been essential to understand the basic principles governing the development of the immune system because most of these mechanisms are conserved in evolution (4–8). However, it is still unclear how these processes are regulated during human development and, in particular, how environmental exposures primarily relevant to the human condition shape individual immune programs in early life. In this review, we summarize our current understanding of mechanisms controlling early life immune development with a focus on antibody production as a key process with sequential molecular checkpoints regulated epigenetically. We then discuss the novel paradigm of human immunology as a complex field that nowadays requires a longitudinal systems-level biology focus, including the study of epigenetic variation and changes during the first few years of life.

## Early Life Antibody Production as a Model to Understand the Basic Principles Governing the Development of the Immune System

Throughout pregnancy, a diverse range of molecules with immune-stimulatory potential such as cells, alloantigens, immune factors, and substances in the amniotic fluid are transferred to the fetus and exposure to trace microbes and microbial antigens can occur *in utero* (9, 10). Nonetheless, antibody responses greatly differ from those during extrauterine life (11, 12). The ability to class-switch from IgM to IgG, IgA, or IgE begins early in fetal life (13). For instance, the fetus contains B-cells primed to IgE as early as 8 weeks and can generate endogenous IgE by 20 weeks of gestation (11). However, only IgM and small amounts of class-switched antibodies are produced *in utero* (11, 13, 14). As a result, newborns rely heavily on protection from maternally transferred antibodies for their transition from the womb to the external world (11–13).

Human babies face the challenge of being born producing only small amounts of class-switched antibodies and must rapidly assemble their own antibody-producing machinery and develop humoral immunocompetence before maternal antibodies disappear, which usually occurs within the first 3–6 months of life (12, 15). This process must have a robust stereotypical program to ensure immunocompetence in infancy to protect against life-threatening infections (15, 16). At the same time, early life antibody production in humans must have plasticity to allow adaptability. The latter is essential to maintain the ability to generate antibody repertoire diversity to face new environments and emergent pathogens. As a result, antibody production represents an ideal evolutionary conserved model to understand the balance between a pre-defined molecular program encoding the stereotypical development of the immune system and the dynamic epigenetic fine-tuning occurring in response to the postnatal environment. This notion has been demonstrated in a recent longitudinal study in which age, geographic location and anemia influenced the composition and dynamics of peripheral immune cells in infants and young children (17).

**Figure 1:**
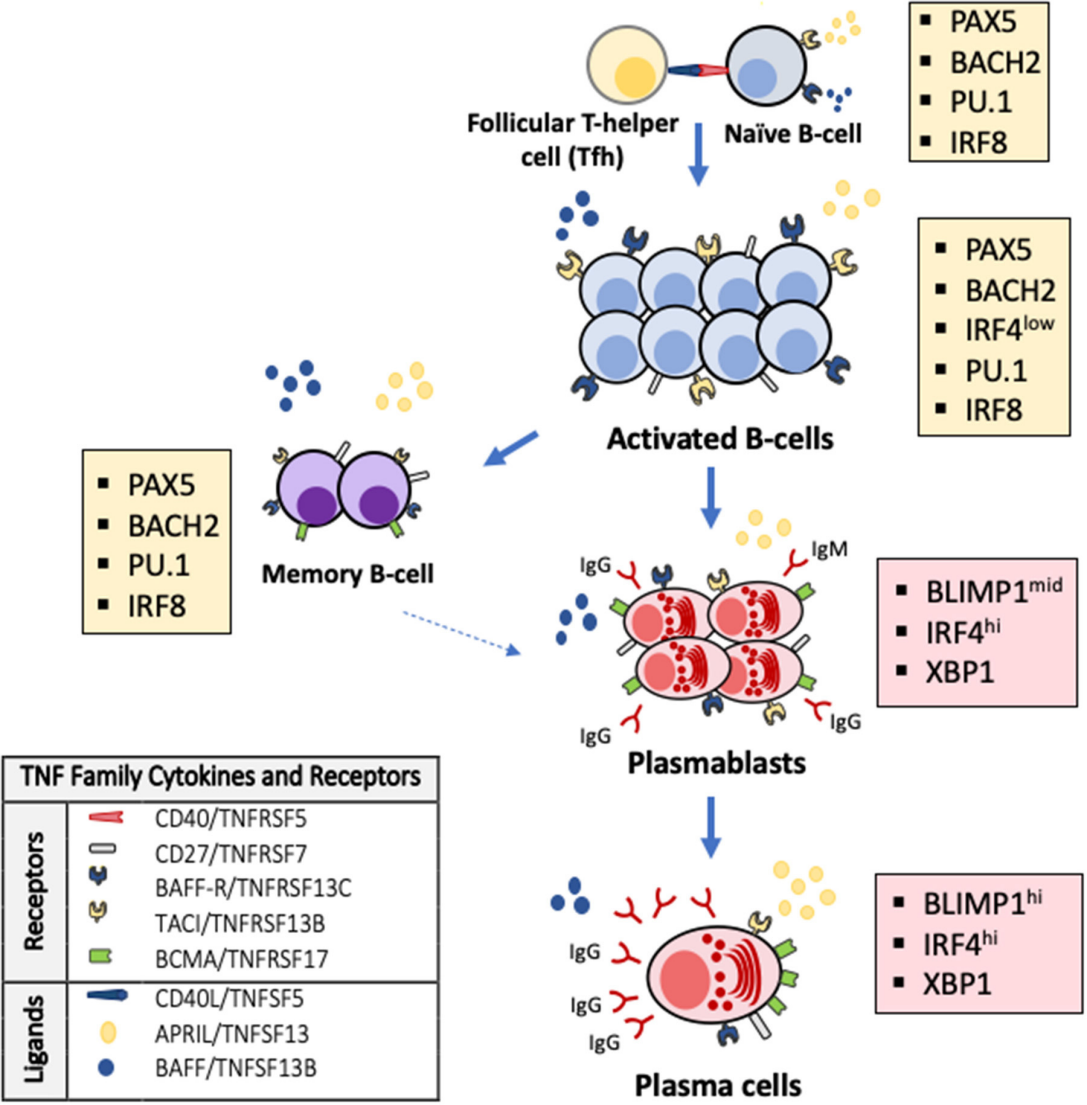
TNF superfamily signaling and transcriptional changes regulate terminal B-cell maturation and antibody production. Activated B-cells undergo apoptosis as default fate unless survival signals are delivered by the TNF superfamily cytokines BAFF (B-cell activation factor, TNFSF13B) and APRIL (a proliferation inducing ligand, TNFSF13) through their receptors TACI (transmembrane activator and calcium modulator and cyclophilin ligand interactor, TNFRSF13B) and BAFF-R (B-cell activating receptor, TNFRSF13C) on the surface of B-cells and BCMA (B-cell maturation antigen, TNFRSF17) on the surface of antibody secreting cells and some memory B-cells. Antibodies are exclusively secreted by ASC that arise from B-cells after profound changes in their transcriptional program (colored boxes). The generation of ASC requires that PAX5 and the transcriptional program that maintains B-cell identity (e.g., IRF8, PU.1, BACH2) are silenced and BLIMP-1, the master regulator of ASC differentiation and associated factors (e.g., XBP1, IRF4) are expressed.

Antibody production is linked to the generation and maintenance of antibody secreting cells (ASC) arising from their B cell precursors and has well-known cellular and molecular checkpoints (18, 19). To secrete antibodies, B-cells must mature into ASC, which may be short-lived effectors in early antibody responses (e.g., plasmablasts) or prolonged lifespan plasma cells that produce long-lasting, highly-specific antibodies (Figure 1). Short-lived plasmablasts are produced during T-cell independent or early T-cell-dependent responses. In contrast, long-lived ASC are generated in a complex process triggered by T-helper cells cross-talk with B-cells in the context of CD40L-CD40 molecular interactions. These interactions occur in the follicles of lymphoid organs and trigger immunoglobulin class-switching to produce antibody isotypes (e.g., IgA, IgG, IgE) (20), antibody somatic hypermutation and clonal selection. These processes result in the terminal differentiation of activated B-cells into memory B-cells and high-affinity ASC (21). Thus, early life antibody production requires sequential steps and molecular signals to maintain B-cell survival and drive the progression to ASC. The lack of these developmental signals results in B-cell death, preventing antibody production and the generation of effective long-term immune memory.

## Stereotypical Molecular Program and Epigenetic Fine-Tuning of Early Life Antibody Production

Several studies have contributed to elucidate the molecular program that controls early life antibody production (3, 22–26). This immune developmental program includes cardinal molecules that orchestrate cell-to-cell interactions and transcription factors that integrate these signals and modify cellular phenotypes according to stereotypical maturational stages (16, 27). It is remarkable that most of these critical cell-to-cell interactions are regulated by a handful of related proteins that belong to a single molecular family, the tumor necrosis factor superfamily of cytokines (TNFSF) (Figure 1). The TNFSF is an evolutionarily conserved superfamily of 19 cytokines that bind one or a restricted number of tumor necrosis factor (TNF) receptors (25, 28, 29). The TNF receptor superfamily (TNFRSF) is a group of 29 related members characterized by the ability to bind tumor necrosis factors (TNFs) via an extracellular cysteine-rich domain (28, 29). TNFRSF members are proteins with powerful effects in apoptosis, proliferation, survival, and differentiation, particularly in all major immune cell types (25, 28–30). In the B-cell compartment, it is noteworthy that B-cells and ASC undergo apoptosis as default program unless specific survival signals are delivered by two members of the TNF cytokine superfamily, BAFF (B-cell activating factor, TNFSF13B) and APRIL (a proliferation inducing ligand, TNFSF13) through binding of their receptors TACI (transmembrane activator and calcium modulator and cyclophilin ligand interactor, TNFRSF13B) and BAFF-R (BAFF receptor, TNFRSF13C) on the surface of B-cells during their late maturational stages (29, 31, 32). Another BAFF/APRIL receptor, BCMA (B-cell maturation antigen, TNFRSF17), located almost exclusively on the surface of ASC, is essential for their survival and, consequently, for the production of long-lived antibodies (Figure 1) (33, 34). Notably, developmental differences are described in the cell expression of B-cell and ASC survival receptors. Specifically, the cell surface expression of TACI, BAFF-R, and BCMA is lower in cord blood B-cells compared with B-cell from adults (35). TNF superfamily receptors and ligands involved in a broader range of cellular responses also signal critical steps during immune development and antibody production. For instance, cell-to-cell interactions mediated by CD40/TNFRSF5 trigger for B-cell class-switch recombination and GC reactions. Differences in gene expression of CD40 and CD40L in B- and T-cells, respectively, have been noted between newborns and adults and lower production of IgA and IgG in response to CD40L stimulation is described in neonatal B-cells, particularly in preterm infants. Other receptors such as CD27/TNFRSF7 participate in T-cell activation, T- and B- cell crosstalk and generation of memory and OX40/TNFRSF4 regulates T-cell survival (25, 30). Monogenic defects in the gene encoding TWEAK (TNF-like weak inductor of apoptosis, TNFSF12) may impair IgA/IgM production and anti-vaccine responses. Pro-apoptotic signals delivered by ligands such as Fas ligand/TNFSF6 or TRAIL/TNFSF10 are critical for lymphocyte selection and development of lymphoid tissues (24, 36). Others, like lymphotoxin β/TNFSF3, a membrane-bound LTα and β complex, regulate GC formation and during early life, may imprint mucosal IgA responses and ASC generation (22). The generation of ASC also involves specific intracellular changes encoded in a transcriptional program driven by master transcription factors of ASC differentiation (18, 19, 37). Specifically, the generation of ASC phenotypes requires the activation of BLIMP1, XBP1, and IRF4, which are repressed during early B-cell development. Conversely, PAX5 and associated transcription factors (e.g., PU.1, IRF8, and BACH2), which are required to maintain B-cell identity, have to be silenced to have cellular differentiation into ASC and allow antibody production (Figure 1) (18, 19, 21, 37, 38).

In summary, antibody production is controlled by a stereotypical developmental program that includes cardinal molecules that drive cell-specific proliferation, survival, and differentiation (e.g., TNFSF and TNFRSF) and specific transcription factors. Changes in these critical checkpoints can potentially shape the development of the immune system and allow dynamic epigenetic fine-tuning occurring in response to the environmental influence during early life. In support of this notion, changes in DNA methylation and histone post-translational modifications have been identified to accompany the formation of GC and ASC generation during immune responses (39–41), and DNA methyltransferases and histone-modifying complexes mediate epigenome changes implicated in ASC differentiation (39–41). Several microRNAs, another group of post-transcriptional regulators of gene expression, converge to modulate class-switch recombination and somatic hypermutation, to upregulate BLIMP1 and IRF4 and to repress BACH2 and PAX5 (42–47), critical steps in ASC differentiation. In different contexts, there is also evidence that TNF superfamily cytokines actions in the immune system are regulated by epigenetic modifications (48–51). Thus, there is compelling evidence demonstrating that epigenetic modifications are crucial for ensuring the generation of ASC and effective antibody production.

## New Insights Into the Stereotypical Early Life Development of the Human Immune System: Values of Birth Cohorts and Longitudinal Systems-Level Analyses

**Figure 2:**
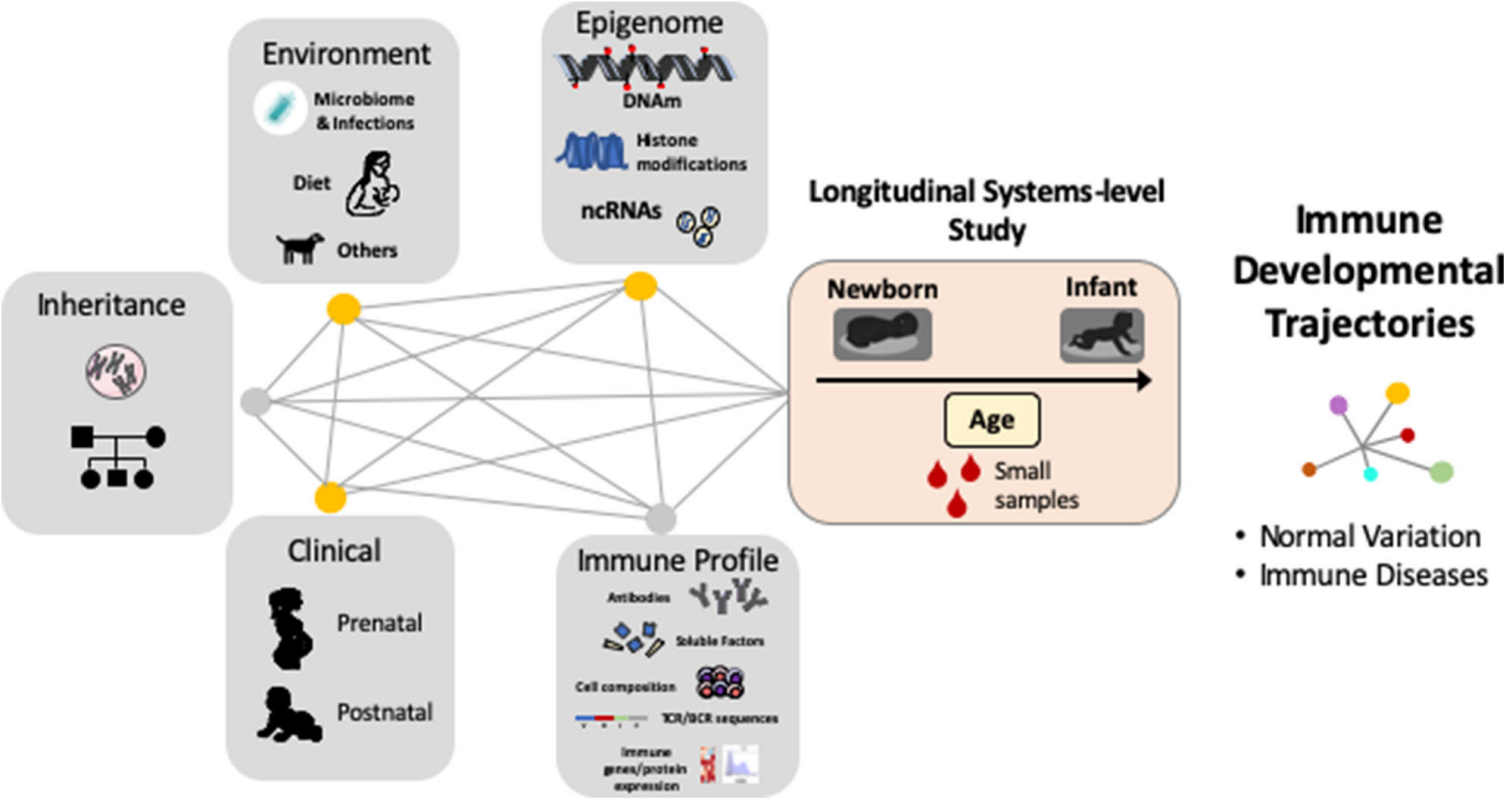
Complex gene, environment and epigenetic interactions shape the human immune system during early life. Systems immunology in longitudinal human-based studies is needed to uncover the “master plan” of human immune development and their relationship with immune diseases. DNAm = DNA methylation, ncRNA = non-coding RNAs, TCR = T-cell receptor, BCR = B-cell receptor.

Despite the fact that early life antibody production has distinct features in humans (12, 15, 23), and that early exposures and consequent shaping of B-cells and ASC identity and function are unique to human infants (22, 52), most research in this field has focused on animal models or human adults and many aspects of how antibody generation is established and occurs during the first a few years of life are not fully understood. However, in recent years several new technological approaches have shown great potential to move forward the emerging field of human systems immunology (53, 54). Indeed, comprehensive immunological analyses are now possible using only small blood samples in human babies and robust computational tools can process and integrate multi-dimensional immunological parameters (12, 16, 52–54). As a result, we are beginning to see how multi-disciplinary scientific collaborations (obstetricians, pediatricians, basic science researchers, and computational biologists) are resulting in the development of new human-based studies that include comprehensive longitudinal systems-level analyses to uncover the “master plan” of the early human development of the immune system (Figure 2).

One of the major milestones of this novel integrative approach is the discovery that the immune system has a stereotypic pattern of development in humans (16). This notion was established by Olin et al. in a study that encompassed longitudinal analyses in 100 newborn children, sampled up to 4 times during their first 3 months of life, including the quantification of 58 immune cell populations by mass cytometry and 267 plasma proteins by immunoassays (16). The result of this study showed that children of different levels of maturity and postnatal environmental conditions converge on a shared developmental trajectory early in life. Interestingly, cord blood immune phenotypes were highly diverse but converged onto a shared developmental path during the first weeks of life, suggesting that there is a “developmental window” during early life for the establishment of critical and long-lasting postnatal immune signatures in human babies.

The notion that there is an early-life developmental window for the environmental imprinting of the human immune system has also been supported by other birth cohort studies. The Canadian Healthy Infant Longitudinal Development (CHILD) cohort included 319 newborns and showed that infants at risk of asthma exhibited transient gut microbial dysbiosis during the first 100 days of life. Specifically, the investigators identified a protective role played by four specific bacterial genera: Faecalibacterium, Lachnospira, Veillonella, and Rothia (55). These data are in agreement with seminal work derived from the Copenhagen Prospective Study on Asthma in Childhood birth cohort (COPSAC) in Denmark (56, 57). The COPSAC cohort demonstrated for the first time that in newborns (*n* = 321), the nasal bacterial colonization with S. pneumoniae, M. catarrhalis, H. influenzae, or a combination of these organisms, is significantly associated with an increased risk for recurrent wheeze and asthma early in life (58). Similar findings have been reported by three additional birth cohorts that have used microbiome and/or host transcriptomic approaches to establish a longitudinal link between the early-life nasal microbiome and the subsequent risk of respiratory diseases (56, 59–61). It is important to note that the latter human-based data are consistent with the substantial collection of accumulated evidence in animal models showing that specific microbial populations during early development can lead to immune-related abnormalities later in life (17, 22, 62–66). Taken together, all these longitudinal systems-level analyses from diverse birth cohorts provide strong support to the concept that environmental exposures taking place during the first weeks of life have a critical influence on the stereotypical development of the immune system in humans.

## Individual Epigenome in Early Life Shapes the Development of the Immune System

The importance of individual epigenetic influences in the development of the human immune system has been established using longitudinal systems-level analyses in twin studies. Brodin et al. conducted a study that included comprehensive measurements of immune cell populations, cytokine responses, and serum proteins in 210 healthy twins between 8 and 82 years of age recruited from the Twin Research Registry at SRI International (67). This seminal work demonstrated that almost all variation in the measured parameters (>80% of variance) is determined by non-heritable influences, which became more pronounced with age, suggesting a cumulative influence of individual epigenetic modifications induced by diverse environmental exposures across the life span. In further considering the implication of these observations, it is worth mentioning that while longitudinal systems-level analyses have established temporal patterns that imply early epigenetic influences in the development of the immune system, the specific epigenetic modifications mediating these effects are still unclear. In this regard, it is noteworthy that DNA methylation is an important mechanism mediating gene-environmental interactions and epigenetic modifications in humans (68, 69) and at the cellular level, DNA methylation is highly sensitive to the cell microenvironment. For example, nutrient availability alters DNA methylation via chromatin-modifying enzymes whose activity is dependent on metabolites such as acetyl-coenzyme A, S-adenosylmethionine, and NAD+ (70, 71). The interplay between metabolites and the microbiota also participates in the DNA methylation process. For instance, *Lactobacillus* produce methyl donors (e.g., folate) required for DNA methylation (70, 72), whereas other bacteria (e.g., *Clostridium)* may induce gene demethylation through metabolites (e.g., butyrate) that downregulate DNA methyltransferases (73).

The DNA methylome undergoes widespread changes during prenatal development and cell differentiation. Indeed, DNA methylation plays a pivotal role in X-chromosome inactivation, genomic imprinting, and long-term gene silencing (74–78). Importantly, in the immune system, DNA methylations critically regulate the early development of hemopoietic progenitors as well as the maturation and lineage commitment of immune cells (79–84). These changes are likely to be influenced by internal and environmental signals that govern B-cell maturation (39). Nevertheless, the interplay between DNA methylations, environmental cues and immune system development during early human life remains largely understudied and poorly understood.

The interaction between early exposures and epigenetic signatures in humans has mostly been studied in birth cohorts using epigenome-wide association analyses (EWAS) (85–91). EWAS data derived from a subset of children in the Boston Birth Cohort (BBC), one of the largest and longest birth cohorts in the U.S. (92), identified that individual epigenetic variations are largely established *in utero*, and that DNA methylation levels in blood cells are very stable within the first 2 years of life (93). Notably, data from the BBC also revealed that the small subset of CpG sites demonstrating significant epigenetic variations during early post-natal life (<1%) were associated with genes involved in the development and function of the immune system (93). Similarly, a recent EWAS study from a large Asian birth cohort of 1,019 infants (68 preterm, 951 full-term) found that the top most statistically significant epigenetic changes from cord blood in premature babies were immune-related genes (94). Another birth cohort in Finland that examined the early-life dynamics of DNA methylation in serial blood samples demonstrated that epigenetic changes in leukocytes during early childhood include several susceptibility loci for immune-related diseases and genes encoding histone modifiers and chromatin remodeling factors that may regulate immune functions (95). Collectively, these studies provide overall support to the concept that the intrauterine and early life environments shape the postnatal program of the human immune system development via epigenetic regulatory mechanisms that include DNA methylation. Currently, although DNA methylation studies have limitations (e.g., reliance upon accessible tissues, confounding by cell heterogeneity), they have been a useful approach to study epigenetic signatures of childhood immune diseases, including allergies and autoimmunity (96–100). Indeed, novel DNA methylation computational analysis can now be employed to trace normal and aberrant hematopoietic cell differentiation (39, 80, 82, 83, 101–103). Because of the correlation of DNA methylation marks with immune cell development, they also have been proposed as an alternative for the diagnosis of immune disorders (104). We believe that in the future, EWAS data may also be used to elucidate relevant epigenomic marks regulating immune system development in humans. The latter will require the multi-disciplinary integration of prospective birth cohorts with scientific teams with expertise in epigenomics systems-level data analyses and the basic mechanisms controlling the cellular and molecular machinery of the different components of the immune system.

## Future Directions

Systems immunology and epigenomics are emerging fields that may greatly advance our current understanding of human immunology during health and disease (12, 54, 105, 106). Longitudinal birth cohort studies that combine cutting-edge multidimensional approaches in epigenetics and immunology are needed to establish the timing, critical checkpoints and early exposures determining non-heritable variability in specific bone marrow-derived and peripheral immune cell populations and functions as well as immune responses in epithelial cells and other key components of the mucosal barriers in humans. In addition, longitudinal systems-level analysis of existing EWAS data from birth cohorts, with a dedicated focus on developmental immunology, may provide novel insights into the epigenetic and molecular control of the stereotypic development of the human immune system. For instance, the epidemiological correlation and functional validation of epigenetic modifications mapped to critical molecular checkpoints for ASC generation (e.g., TNF superfamily signaling genes, BLIMP1, XBP1, and IRF4) can provide new clues on how the intrauterine and early post-natal human environment modulates antibody production and elucidate new mechanisms and pathways associated with the development and maintenance of long-term protective responses to immunizations and against pathogens.

In summary, we believe that the challenge for the next generation of scientists in the field of human immunology will be to integrate the basic principles governing the development of the immune system with the increasingly large multidimensional data (including epigenetic) and clinical evidence, derived from prospective human birth cohorts during health and disease. More sophisticated tools for immune phenotyping using small-volume samples and increasingly complex systems biology analytical approaches offer exciting opportunities to simultaneously interrogate clinical, genetic, epigenetic and functional signatures of immune cell populations in newborns and young children. We anticipate that the progress in epigenomics during the next few years may lead to the discovery of fundamental early-life gene-environmental factors determining the development of protective immune responses in humans, and its relation with the risk and resilience to develop a myriad of immune-related disorders during infancy and across the life span.

## Author Contributions

MG and GN wrote the manuscript. XH, GN, and XW provided direction, revised it for important intellectual content, and provided critical feedback. All authors contributed to the article and approved the submitted version.

## Conflict of Interest

The authors declare that the research was conducted in the absence of any commercial or financial relationships that could be construed as a potential conflict of interest.
